# Down-regulation of lncRNA snaR is correlated with postoperative distant recurrence of HPV-negative cervical squamous cell carcinoma

**DOI:** 10.1042/BSR20181213

**Published:** 2018-11-14

**Authors:** Zhenli Zheng, Yuqing Gao

**Affiliations:** Department of Gynecology, Zhoukou Central Hospital, Zhoukou City, Henan Province, 466000, PR. China

**Keywords:** HPV-negative cervical squamous cell carcinoma, invasion, lncRNA snaR, migration, TGF-β1

## Abstract

Long non-coding RNAs (lncRNAs) snaR is a newly identified lncRNA with known functionality only in colon cancer. Our study was carried out to investigate the involvement of lncRNA snaR in human papillomaviruses (HPV)-negative cervical squamous cell carcinoma (CSCC). In the present study, plasma levels of lncRNA snaR in 108 patients with HPV-negative CSCC at stage I and II, and 35 healthy female controls were detected by real-time quantitative PCR. ROC curve analysis was performed to evaluate the diagnostic value of lncRNA snaR for HPV-negative CSCC. All patients were subjected to surgical resection and followed-up for 5 years to record cancer recurrence. lncRNA snaR expression vectors were transfected into HPV-negative CSCC cells. Cell migration and invasion ability were evaluated by Transwell migration and invasion assay, respectively. Expression levels of TGF-β1 were determined by Western blot. It was observed that lncRNA snaR was down-regulated in HPV-negative CSCC patients comparing with healthy controls. Down-regulation of lncRNA snaR effectively distinguished HPV-negative CSCC patients from healthy controls. lncRNA snaR was further down-regulated in patients with distant recurrence (DR) but not in patients with local-recurrence or without recurrence. lncRNA snaR overexpression decreased TGF-β1 expression in CSCC cells, while exogenous TGF-β1 treatment showed no significant effects on lncRNA snaR expression. lncRNA snaR overexpression inhibited cancer cell migration and invasion, while TGF-β1 treatment attenuated the inhibitory effect of lncRNA snaR overexpression on cancer cell migration and invasion. We therefore conclude that down-regulation of lncRNA snaR may induce postoperative DR of HPV-negative CSCC possibly through the interactions with TGF-β1.

## Introduction

Surgical resection is a promising treatment strategy for solid tumors at early stages. However, recurrence of tumor is frequently observed after treatment, which significantly shortens patients’ survival time [1,2]. Therefore, accurate prediction of cancer postoperative recurrence is always needed [3,4]. Cervical cancer is a frequently diagnosed malignancy amongst females that causes high modality rate [5]. As one of the major pathological types of cervical cancer, cervical squamous cell carcinoma (CSCC) accounts for more than 80% of the cases [6]. Most cases of CSCC are caused by human papillomaviruses (HPV) infection [7]. At present, the incidence of HPV-positive CSCC has been significantly reduced due to the popularization of HPV infection screening program [8]. However, CSCC can also be caused by other factors. HPV-negative CSCC usually shows more aggressive behaviors and causes worse survival compared with HPV-positive cases [9].

A growing body of research indicates long non-coding RNAs (lncRNAs) are key players in various pathological changes including the development of human cancers [10]. Recent studies also showed that the recurrence of cancers after treatment is usually accompanied with changes in expression patterns of a large set of lncRNAs [11], indicating the involvement of lncRNAs in this processes. TGF-β signaling plays critical roles in both growth and migration of cancer cells [12]. It has been well established that TGF-β signaling under certain conditions achieves its biological roles through the interactions with lncRNAs [13]. lncRNA snaR has recently been reported to be involved in the development of drug resistance in colon cancer cells [14]. In another study, lncRNA snaR was found to play regulatory role in the proliferation, migration, and invasion of triple negative breast cancer cells [15]. However, the involvement of lncRNA snaR in other cancers is unknown. Our preliminary data (microarray) revealed a reverse correlation between TGF-β1 mRNA and lncRNA snaR across HPV-negative CSCC specimens (data not shown). In our study, we observed that lncRNA snaR was down-regulated in HPV-negative CSCC. We also found that lncRNA snaR may participate in the distant recurrence (DR) of HPV-negative CSCC after surgical resection through the interactions with TGF-β1.

## Materials and methods

### Subjects

From January 2010 to 2013, a total of 212 HPV-negative CSCC patients were diagnosed in Zhoukou Central Hospital. Amongst those patients, 108 were included in the present study to serve as research subjects according to inclusion and exclusion criteria. Inclusion criteria: (i) patients diagnosed as HPV-negative CSCC through pathological examinations; (ii) patients at early stages (stage I and II); (iii) patients diagnosed and treated for the first time; (iv) patients completed follow-up; (v) patients willing to participate. Exclusion criteria: (i) patients complicated with other severe diseases; (ii) patients with other cervical diseases; (iii) patients who were treated before admission; (iv) patients died before the diagnosis of recurrence. Age of those patients ranged from 27 to 69 years, with a mean age of 48.6 ± 6.1 years. At the same time, a total of 35 healthy females, who received normal physiological examinations during the same time period, were also included as control group. Age of control group ranged from 28 to 68 years, with a mean age of 48.1 ± 5.3 years. No significant differences in age and gender were found between two groups. The present study got approval from Ethics Committee of Zhoukou Central Hospital. All patients signed informed consent. All experiments were performed in accordance with the World Medical Association Declaration of Helsinki.

### Specimen collection and follow-up

All patients received surgical resection. After discharge, those patients were followed-up for 5 years to record recurrence. Blood was extracted from both patients and healthy controls on the day of admission. Blood was also collected during follow-up on the day of the diagnosis of recurrence or at the end of follow-up. Blood was used to prepare plasma samples using conventional method.

### Real-time quantitative PCR

TRIzol reagent (Invitrogen, U.S.A.) was used for all total RNA extractions. After RNA extraction, RNA samples were quantitated and subjected to reverse transcription using SuperScript III Reverse Transcriptase (Thermo Fisher Scientific) through following thermal conditions: 50°C for 20 min and 75°C for 15 min. All PCR reactions were prepared using SYBR^®^ Green Real-Time PCR Master Mixes (Thermo Fisher Scientific) and following primers: 5′-TGGAGCCATTGTGGCTCCGGCC-3′ (forward) and 5′-CCCATGTGGACCAGGTTGGCCT-3′ (reverse) for snaR; 5′-CAGGAGGCATTGCTGATGAT-3′ (forward) and 5′-GAAGGCTGGGGCTCATTT-3′ (reverse) for GAPDH. PCR reaction conditions were: 95°C for 1 min 10 s, followed by 40 cycles of 95°C for 15 s and 58°C for 40 s. *C*_T_ values were normalized using 2**^−^**^ΔΔ*C*_T_^ methods.

### Cell lines and cell culture

Human CSCC cell line C33A (HPV negative) and normal cervical cell line HCvEpC (HPV negative) were purchased from ATCC (U.S.A.). Cells were cultured in strict accordance with manufacturer’s instructions. PCR was performed to amplify full length lncRNA snaR cDNA. lncRNA snaR cDNA was inserted into pcDNA3.1 vector to construct lncRNA snaR expression vector. Cells were cultured over night to reach 80–90% confluence. Lipofectamine 2000 (11668-019, Invitrogen, Carlsbad, U.S.A.) reagent was used to transfect 50 nM vectors into cancer cells. Untransfected cells were used as control cells. Cells transfected with empty vectors were negative controls cells.

### Transwell migration and invasion assay

Expression of lncRNA snaR was detected by quantitative real-time PCR (qRT-PCR) at 12 h after transfection. Overexpression rate above 200 % was reached. After that, cells were collected and cell suspensions with a cell density of 3 × 10^4^ cells/ml were prepared and *in vitro* cell migration, and invasion abilities were evaluated by Transwell migration and invasion assay. Briefly, the upper chamber was filled with 0.1 ml cell suspension containing 3 × 10^3^ cells, while the lower chamber was filled with RPMI-1640 medium containing 20 % FBS. The chamber was kept at room temperature for 12 h. Membranes were then collected and stained with 0.5% crystal violet (Sigma–Aldrich, U.S.A.) at room temperature for 15 min. The upper chamber was precoated with Matrigel (356234, Millipore, U.S.A.) before cell invasion assay, while the upper chamber was not precoated in migration assay.

### Western blot

Total RNA was extracted using RIPA solution (Thermo Fisher Scientific, U.S.A.). Protein concentrations were measured by BCA assay. After that, protein samples were denatured and 10% SDS/PAGE gel electrophoresis was performed with 30 µg protein in each well. After gel transfer to PVDF membranes, membranes were blocked in 5% skimmed milk at room temperature for 1.5 h, followed by incubation with rabbit anti-human primary antibodies of TGF-β1 (1: 1200, ab92486, Abcam), and GAPDH antibody (1: 1200, ab37168, Abcam) at 4°C overnight. The next day, membranes were further incubated with anti-rabbit IgG-HRP secondary antibody (1:1000, MBS435036, MyBioSource) at room temperature for 2 h. Signal development was performed using ECL (Sigma–Aldrich, U.S.A.). Signal was normalized using ImageJ software.

### Statistical analysis

All data analyses were performed using Graphpad Prism 6 software. All experiments were performed in triplicate manner. Gene expression and cell migration and invasion data were expressed as mean ± S.D. Comparisons between two groups were performed by unpaired *t* test. Before-and-after treatment comparisons within a group were performed using paired *t* test. Comparisons amongst multiple groups were performed by one-way ANOVA and LSD test. *P*<0.05 was considered to be statistically significant.

## Results

### Down-regulation of plasma lncRNA snaR effectively distinguished HPV-negative CSCC patients from healthy controls

**Figure 1:**
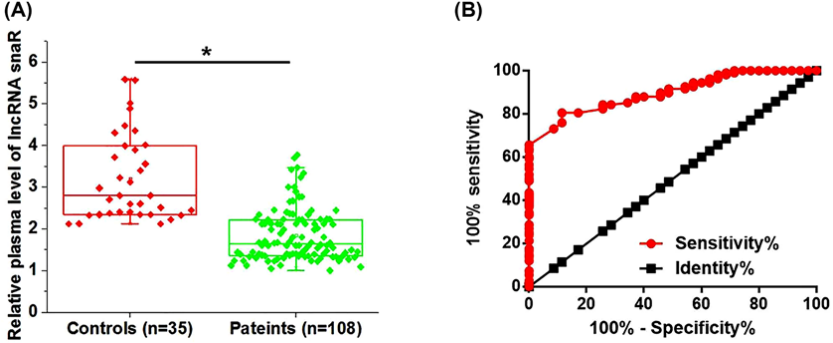
Down-regulation of plasma lncRNA snaR effectively distinguished HPV-negative CSCC patients from healthy controls Plasma lncRNA snaR was significantly down-regulated in HPV-negative CSCC patients than in healthy controls (**A**) and the down-regulation of plasma lncRNA snaR effectively distinguished HPV-negative CSCC patients from healthy controls (**B**) (*P<0.05).

Plasma levels of lncRNA snaR in both HPV-negative CSCC patients and healthy controls were measured by qRT-PCR. As shown in Figure 1A, plasma levels of lncRNA snaR were significantly lower in HPV-negative CSCC patients than those in healthy controls (*P*<0.05). ROC curve analysis was performed to evaluate the diagnostic value of plasma lncRNA snaR for HPV-negative CSCC. As shown in Figure 1B, area under the curve (AUC) was 0.8972, with standard error of 0.02522 and 95% confidence interval of 0.8478–0.9467.

### lncRNA snaR was down-regulated in patients with DR, but not in patients with local-recurrence or without recurrence during follow-up

**Figure 2:**
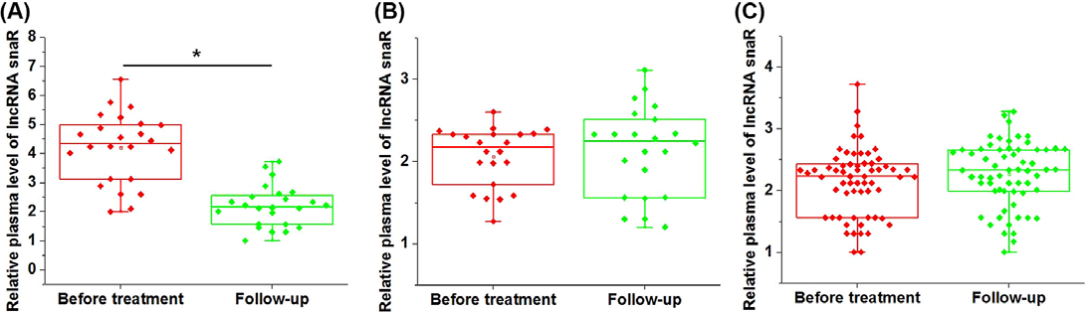
lncRNA snaR was down-regulated in patients with DR but not in patients with local-recurrence or without recurrence during follow-up This figure shows the comparison between pretreatment levels of plasma lncRNA snaR and plasma lncRNA snaR levels during follow-up amongst patients with DR (**A**), patients with LR (**B**) and patients with NR (**C**) (*P<0.05).

After 5-year follow-up, local recurrence (LR) was observed in 22 patients, DR was observed in 24 patients, the rest 62 patients showed no recurrence (NR). As shown in Figure 2, compared with pretreatment levels, plasma lncRNA snaR was significantly down-regulated in patients with DR during follow-up (Figure 2A, *P*<0.05). However, no significant changes were found in patients with LR (Figure 2B, *P*>0.05) or NR (Figure 2C, *P*>0.05).

### lncRNA snaR is a potential upstream activator of TGF-β1 in HPV-negative CSCC

**Figure 3:**
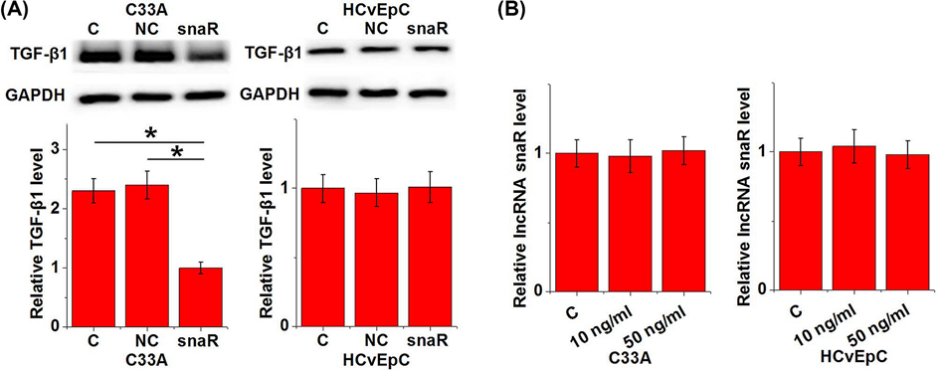
lncRNA snaR is a potential upstream activator of TGF-β1 in HPV-negative CSCC lcRNA snaR overexpression led to significantly down-regulated expression of TGF-β1 in cells of HPV-negative CSCC cell line C33A but not in cells of HPV-negative normal cervical cell line HCvEpC (**A**). In contrast, treatment with exogenous TGF-β1 at doses of 10 and 50 ng/ml showed no significant effects on lncRNA snaR in cells of both cell lines (**B**) (*P<0.05).

The above mentioned data indicate the possible involvement of lncRNA snaR in the DR of HPV-negative CSCC. TGF-β signaling plays a pivotal role in cancer metastasis. Therefore, the potential interaction between lncRNA snaR and TGF-β1 in HPV-negative CSCC was investigated. Compared with control cells (C) and negative control cells (NC), lncRNA snaR overexpression led to significantly down-regulated expression of TGF-β1 in cells of HPV-negative CSCC cell line C33A but not in cells of HPV-negative normal cervical cell line HCvEpC (Figure 3A, *P*<0.05). In contrast, treatment with exogenous TGF-β1 (R&D Systems) at doses of 10 and 50 ng/ml showed no significant effects on lncRNA snaR in cells of both cell lines (Figure 3B).

### lncRNA snaR overexpression inhibited HPV-negative CSCC cell migration and invasion

**Figure 4:**
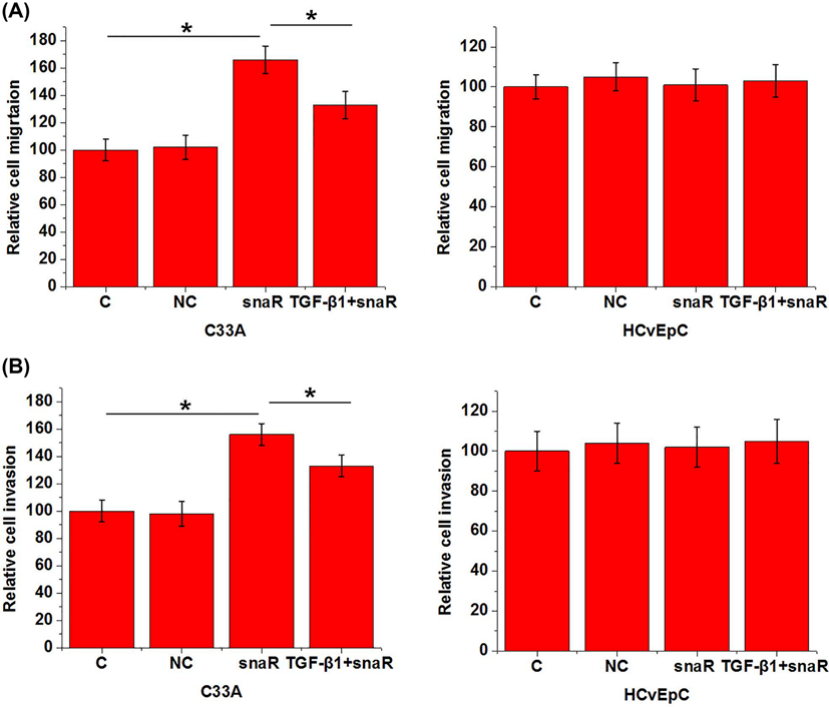
lncRNA snaR overexpression inhibited HPV-negative CSCC cell migration and invasion lncRNA snaR overexpression inhibited HPV-negative CSCC cell migration (**A**) and invasion (**B**), while exogenous TGF-β1 treatment attenuated this effect (*P<0.05).

Transwell migration and invasion assay was performed to investigate the effects of lncRNA snaR overexpression and exogenous TGF-β1 on cancer cell migration and invasion. Compared with control cells (C) and negative control cells (NC), lncRNA snaR overexpression led to significantly promoted cell migration (Figure 4A) and invasion (Figure 4B) of cells of HPV-negative CSCC cell line C33A (*P*>0.05), but not cells of HPV-negative normal cervical cell line HCvEpC. In addition, treatment with exogenous TGF-β1 at a dose of 10 ng/ml attenuated the inhibitory effects of lncRNA snaR overexpression on migration (Figure 4A) and invasion (Figure 4B) of cells of HPV-negative CSCC cell line C33A (Figure 4b, *P*>0.05).

## Discussion

lncRNA snaR has characterized functionality in the development of drug resistance in colon cancer cells and in the proliferation, migration, and invasion of triple negative breast cancer cells [14,15]. The key finding of our study is that lncRNA snaR is likely a tumor suppression lncRNA in HPV-negative CSCC, and the down-regulation of lncRNA snaR mediates the DR after surgical resection.

Differential expression in patients and healthy people indicates the involvement of a certain gene in diseases. In the present study, plasma lncRNA snaR was detected in all patients with HPV-positive CSCC and healthy controls, indicating the role of lncRNA snaR as a circulating signaling molecular in the human body. Although lncRNA snaR has been reported to be involved in colon cancer and triple negative breast cancer cells [14,15], its expression pattern in the human body is still unknown. In our study, plasma levels of lncRNA snaR were found to be significantly lower in HPV-negative CSCC patients than in healthy controls, indicating the potential role of lncRNA snaR as a tumor suppression lncRNA in this disease.

Tumor metastasis is a major cause of deaths amongst cancer patients [16], and early diagnosis and treatment is still critical for the survival [17]. In our study, we only included HPV-negative CSCC patients at stage I and II, which are considered to be the early stages of cancer. ROC curve analysis showed that down-regulation of lncRNA snaR effectively distinguished cancer patients from healthy controls, indicating that lncRNA snaR may serve as a potential diagnostic marker for HPV-negative CSCC. It is also worth to note that the expression pattern of lncRNA snaR is still unknown in other diseases. Therefore, lncRNA snaR should be combined with other diagnostic markers to improve the diagnostic specificity.

Local and DRs are two types of cancer recurrence. Compared with LR, survival conditions of patients with DR are even worse [18]. Treatment of CSCC is always followed by the high recurrence rate [19]. Although several indicators have been reported to have predictive values for the recurrence of CSCC, accurate and early prediction is still challengeable [20]. In our study, we observed significantly down-regulated plasma levels of lncRNA snaR only in patients with DR, but not in patients with LR or patients without recurrence, indicating the potential involvement of lncRNA snaR specifically in DR amongst HPV-negative CSCC patients undergoing surgical resection.

TGF-β signaling is not only involved in the development and progression of cancers [12], but also participates in cancer recurrence [21]. Our study suggested that lncRNA snaR was likely a upstream inhibitor of TGF-β signaling in HPV-negative CSCC because lncRNA snaR overexpression led to down-regulated TGF-β1 expression in HPV-negative CSCC cells, while exogenous TGF-β1 treatment showed on significant effects on lncRNA snaR. In addition, exogenous TGF-β1 treatment also attenuated the inhibitor effect of lncRNA snaR overexpression on cancer cell migration and invasion. Therefore, down-regulation of lncRNA snaR may mediate the DR of HPV-negative CSCC through the interactions with TGF-β1. It is also worth to note that lncRNA snaR overexpression showed no significant effects on normal cervical cells. Therefore, lncRNA snaR may serve as a potential therapeutic target for HPV-negative CSCC. It also suggests that that the regulatory effect of lncRNA snaR on TGF-β1 expression is indirect and disease-related factors may be involved.

In conclusion, lncRNA snaR was down-regulated in HPV-negative CSCC. Down-regulation of lncRNA snaR may mediate the DR of HPV-negative CSCC through the interactions with TGF-β1.

The research has been carried out in accordance with the World Medical Association Declaration of Helsinki.

## Abbreviations

CSCC: cervical squamous cell carcinoma
HPV: human papillomavirus
lncRNA: long non-coding RNA
LR: local recurrence
NR: no recurrence
qRT-PCR: quantitative real-time PCR
